# Time-resolved pair distribution function analysis of disordered materials on beamlines BL04B2 and BL08W at SPring-8

**DOI:** 10.1107/S1600577518011232

**Published:** 2018-09-26

**Authors:** Koji Ohara, Satoshi Tominaka, Hiroki Yamada, Masakuni Takahashi, Hiroshi Yamaguchi, Futoshi Utsuno, Takashi Umeki, Atsushi Yao, Kengo Nakada, Michitaka Takemoto, Satoshi Hiroi, Naruki Tsuji, Toru Wakihara

**Affiliations:** a Research and Utilization Division, Japan Synchrotron Radiation Research Institute, 1-1-1 Kouto, Sayo, Hyogo 679-5198, Japan; b Synchrotron X-ray Station at SPring-8, National Institute for Materials Science (NIMS), 1-1-1 Kouto, Sayo, Hyogo 679-5148, Japan; c International Center for Materials Nanoarchitectonics (WPI-MANA), National Institute for Materials Science (NIMS), 1-1 Namiki, Tsukuba, Ibaraki 305-0044, Japan; d Department of Chemical System Engineering, University of Tokyo, 7-3-1 Hongo, Bunkyo-ku, Tokyo 113-8656, Japan; e Department of Interdisciplinary Environment, Graduate School of Human and Environmental Studies, Kyoto University, Yoshida-nihonmatsu, Sakyo, Kyoto 606-8501, Japan; f Advanced Technology Research Laboratories, Idemitsu Kosan Co. Ltd, 1280 Kamiizumi, Sodegaura, Chiba 299-0293, Japan

**Keywords:** time-resolved measurements, pair distribution functions, beamlines, disordered materials, crystallization

## Abstract

A dedicated apparatus has been developed for studying structural changes in amorphous and disordered crystalline materials substantially in real time. The apparatus, which can be set up on beamlines BL04B2 and BL08W at SPring-8, mainly consists of a large two-dimensional flat-panel detector and high-energy X-rays, enabling total scattering measurements to be carried out for time-resolved pair distribution function (PDF) analysis in the temperature range from room temperature to 873 K at pressures of up to 20 bar. For successful time-resolved analysis, a newly developed program was used that can monitor and process two-dimensional image data simultaneously with the data collection. The use of time-resolved hardware and software is of great importance for obtaining a detailed understanding of the structural changes in disordered materials, as exemplified by the results of commissioned measurements carried out on both beamlines. Benchmark results obtained using amorphous silica and demonstration results for the observation of sulfide glass crystallization upon annealing are introduced.

## Abstract 2

A new apparatus for total scattering measurements has been developed with a variable camera length using a 16-inch two-dimensional flat-panel detector to observe structural changes in amorphous and disordered crystalline materials substantially in real time on beamlines BL08W and BL04B2 at SPring-8. This paper presents the successful time-resolved pair distribution function analysis at SPring-8, and a newly developed program for data conversion from two-dimensional images into one-dimensional total scattering patterns.

## 1. Introduction   

Pair distribution function (PDF) analysis of total scattering data covering a wide range of momentum transfer (*Q*), particularly up to a sufficiently large momentum transfer measured using high-energy X-rays available from synchrotron radiation, is widely utilized in the structural study of amorphous materials and disordered crystalline materials (Billinge & Levin, 2007 ▸; Young & Goodwin, 2011 ▸; Keen & Goodwin, 2015 ▸). From total scattering measurements, quantitative information on atomic arrangements, namely the relationship between interatomic distances, *r*, and atomic pair densities, *i.e.* the PDF, *g*(*r*), can be calculated from the experimentally accessible function of the reduced PDF, *G*(*r*), as follows:
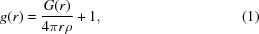
where ρ is the average number density of atoms. *G*(*r*) is calculated as follows by Fourier transformation of the total structure factor, *F*(*Q*), which is a normalized form of the total scattering data and represents the oscillation associated with the density distribution in a material:
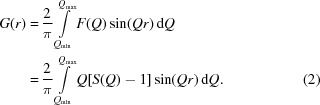
Because this Fourier transform results in an *r* spacing of π/*Q*_max_, *S*(*Q*) data measured up to a higher *Q* region result in PDFs with a higher real-space resolution. For example, high-energy X-rays of over 40 keV (or a shorter photon wavelength of λ < 0.31 Å) enable us to obtain high momentum transfers, *Q* > 20 Å^−1^, which also depend on the scattering angle, 2θ (*Q*λ = 4πsinθ).

Because the physical meaning of *G*(*r*) might not be straightforward, a total correlation function, *T*(*r*), is often used to discuss the structural information (Keen, 2001 ▸),
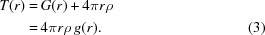
A peak in *T*(*r*) indicates the existence of atoms with a density exceeding the average number density at a distance *r*, whereas a valley suggests the absence of atoms. PDF analysis with high-energy X-rays has been reviewed in detail by Benmore (2012 ▸).

Beamline BL04B2 at SPring-8 was designed for high-energy X-ray diffraction measurements to perform high-resolution X-ray PDF analysis (Isshiki *et al.*, 2001 ▸; Kohara *et al.*, 2007 ▸), contributing to scientific research on the structural study of disordered materials (Matsunaga *et al.*, 2011 ▸; Ohara *et al.*, 2012 ▸; Akola *et al.*, 2013 ▸; Kohara *et al.*, 2014 ▸; Liu *et al.*, 2016 ▸; Umeda *et al.*, 2017 ▸; Onodera *et al.*, 2017 ▸). One measurement for PDF analysis on BL04B2 takes at least 2–3 h, owing to the necessity of scanning a point detector with an energy window such as a Ge-SDD to eliminate the higher harmonic contamination of X-rays. This long measurement due to the use of scanning point detectors has been typical at SPring-8, hampering observations of rapid structural changes in dis­ordered materials. For example, observations of structural changes upon the growth or ageing of disordered materials, which may occur within a few minutes, are required to understand the roles of such processes. Understanding the structural relaxation mechanism in amorphous materials, including organic materials such as pharmaceutical molecules (Tominaka *et al.*, 2017 ▸), and the crystallization mechanism of glasses during annealing treatment are of great interest. Nowadays, such time-resolved PDF analysis is being utilized at other synchrotron facilities, such as the APS (Chupas *et al.*, 2003 ▸, 2004 ▸, 2007 ▸; Kramer, 2007 ▸; Jensen *et al.*, 2012 ▸), the ESRF (Abdala *et al.*, 2014 ▸; Terban *et al.*, 2015 ▸) and the DLS (Keen *et al.*, 2018 ▸), using two-dimensional amorphous silicon (a-Si) digital X-ray flat-panel detectors (FPDs). This is because FPDs possess characteristics suitable for time-resolved PDF analysis, such as high efficiency for the detection of high-energy X-rays, a high dynamic range and contrast, and a high throughput with a frame rate of 15 frames s^−1^.

To achieve such time-resolved PDF analysis at SPring-8, we have developed a new apparatus for total scattering measurements with a variable camera length using a 16-inch two-dimensional FPD (XRD1621 CN3), which we tested on beamlines BL04B2 and BL08W. The sample environment is controlled using a variety of sample stages. For example, (i) solvothermal/hydrothermal conditions are realized using a microwave reactor (Initiator from Biotage) in the temperature range from room temperature to 573 K at a pressure of less than 20 bar (1 bar = 100 000 Pa; Tominaka *et al.*, 2018 ▸), and (ii) high-temperature conditions up to 873 K are realized by a heating cell. In addition to being designed for *in situ* measurements, the apparatus used for time-resolved PDF analysis is compatible with the existing facilities on BL04B2, such as an aerodynamic levitation furnace, an acoustic levitation furnace and a diamond anvil cell. In this paper, we present the design and experimental verification of the new apparatus for time-resolved PDF analysis.

## 2. PDF analysis on high-energy X-ray total scattering beamlines at SPring-8   

Beamlines BL04B2 and BL08W have been widely used in structural studies of disordered materials over the past 20 years (Ohno *et al.*, 2001 ▸; Kohara *et al.*, 2004 ▸, 2014 ▸; Inui *et al.*, 2007 ▸; Matsunaga *et al.*, 2011 ▸; Li *et al.*, 2018 ▸). Total scattering measurement (or PDF analysis) using high-energy X-rays is useful for quantitatively determining the local structure of disordered materials at low scattering angles with a wide *Q* range. BL04B2 is equipped with two Si crystals as a monochromator, which provides fixed-energy X-rays of 37.7 keV from Si(111), 61.4 keV from Si(220) and 113.1 keV from Si(111) (third-harmonic generation). The energy of 61.4 keV is mainly used in the PDF analysis. On the other hand, BL08W can provide fixed-energy X-rays of 115 keV using Si(440). The energy resolutions Δ*E*/*E* of BL04B2 and BL08W are approximately 5 × 10^−3^ and 1 × 10^−3^, respectively. The beam dimensions at the sample position are 1.0 mm (vertical) × 1.0 mm (horizontal). The photon flux on BL08W is approximately 100 times higher than that on BL04B2, at 1.0 × 10^13^ photons s^−1^. Further details of these beamlines are available in the literature (Kohara *et al.*, 2007 ▸; Marechal *et al.*, 1998 ▸). On BL04B2, a total scattering apparatus consisting of point detectors installed along the flight path to eliminate the harmonic contamination of X-rays is continuously available. This system is mainly used for *ex situ* study of disordered materials. On the other hand, on BL08W, a temporary apparatus has been installed to enable the use of X-rays with higher energy or flux to obtain total scattering data from samples containing a heavy element (Yoneda *et al.*, 2013 ▸; Kohara *et al.*, 2014 ▸) or from samples in an unstable environment subjected to aerodynamic (Kohara *et al.*, 2014 ▸) or acoustic levitation.

**Figure 1:**
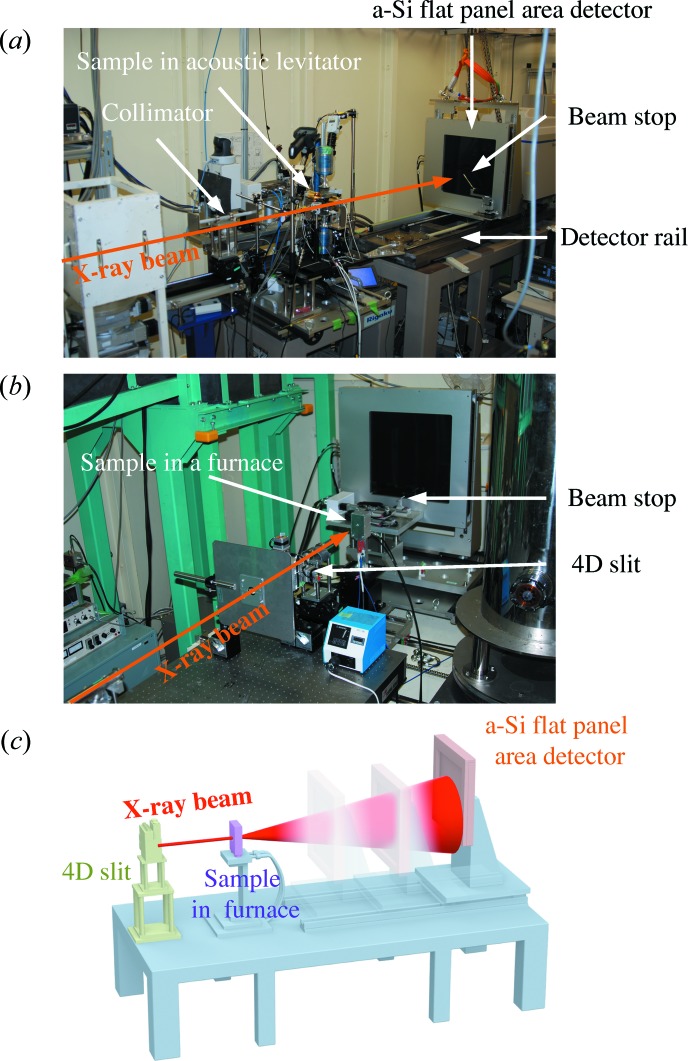
Photographs and an illustration of the experimental setups of the flat-panel area-detector apparatus for the analysis of disordered materials. (*a*) High-resolution mode on BL04B2 with an acoustic levitator. (*b*) High-*Q* measurement mode on BL08W with a furnace. (*c*) A schematic drawing of the apparatus used for *in situ* PDF analysis.

**Figure 2:**
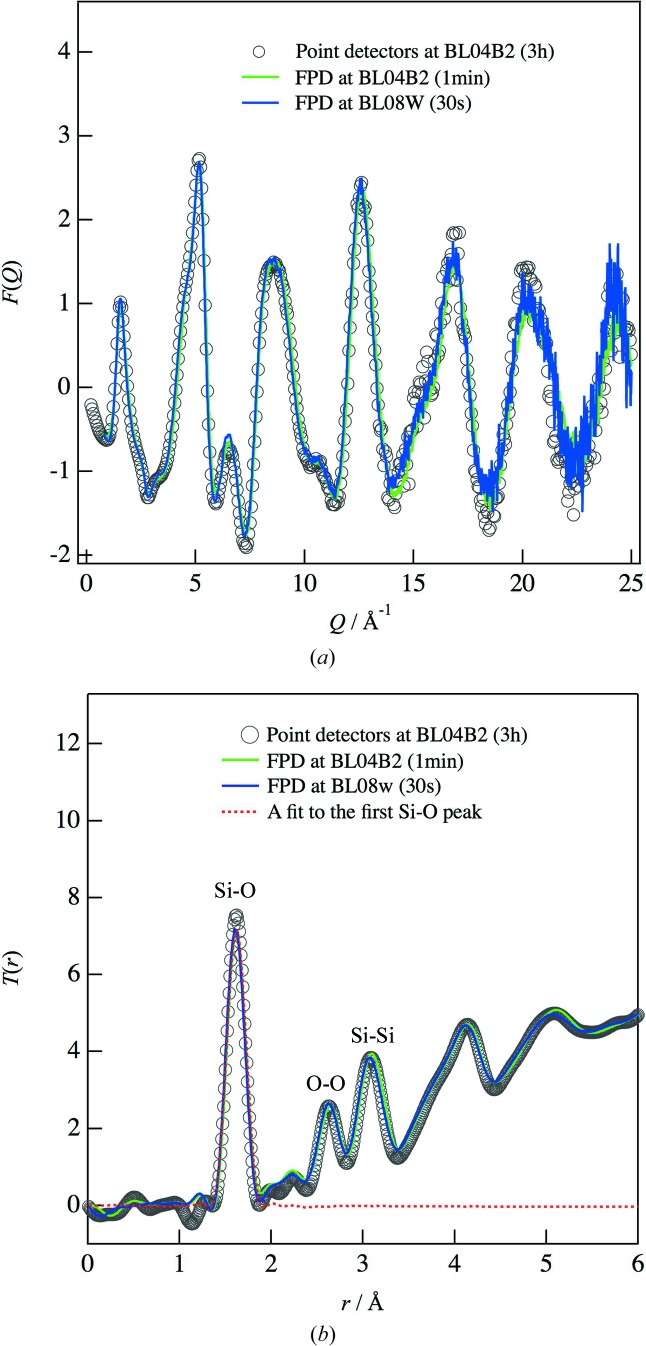
(*a*) Total structure factors *F*(*Q*) and (*b*) total correlation function *T*(*r*) of amorphous silica obtained on BL04B2 and BL08W using the FPD (1 min), compared with those obtained on BL04B2 using point detectors (3 h). Fourier transforms were carried out for the data for a constant *Q*_max_ = 26 Å^−1^ using the Lorch function (Lorch, 1969 ▸). A fit to the first Si—O peak in the X-ray correlation function (Mozzi & Warren, 1969 ▸) is also shown by the red dashed line.

We have installed a large flat-panel area detector on both beamlines, as illustrated in Fig. 1 ▸. The X-ray diffraction measurements on these beamlines have different features, particularly for the photon energy and flux as described above, which in turn result in different resolutions in real/reciprocal space, as summarized in Table 1 ▸. For example, the camera length can be changed from 372 mm up to 866 mm on BL08W, which corresponds to *Q* = 50–25 Å^−1^ (d*Q* = 0.04–0.02 Å^−1^). This difference in the specifications does not result in an obvious difference in the PDF data, at least in the standard data for amorphous silica, as shown in Fig. 2 ▸. The figure shows that the oscillations in *F*(*Q*) do not decay up to the high-*Q* region, as is always the case with Si—O covalent bonds. Thus, it is suitable for verifying measurements. Regarding *F*(*Q*), compared with the data obtained using six point detectors on BL04B2, the total scattering data collected using the FPD are identical, at least in the observed range of *Q* (up to 25 Å^−1^).

**Figure 3:**
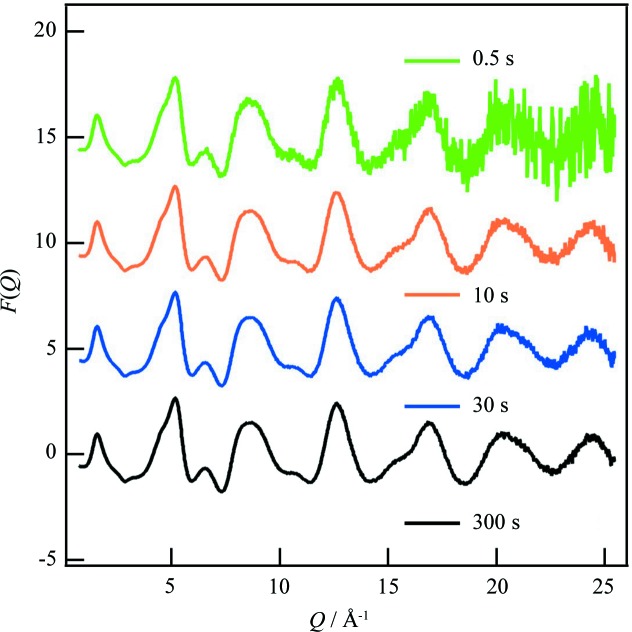
Total structure factors *F*(*Q*) of amorphous silica on BL04B2. The data were collected with integration periods of 0.5, 10, 30 and 300 s.

The total correlation functions, *T*(*r*), for the amorphous silica were obtained from *F*(*Q*) at intervals of 0.05 Å^−1^ in the *Q* range up to 25 Å^−1^ and are shown in Fig. 2 ▸(*b*). The data obtained using the FPD are similar, have a sufficient real-space resolution and exhibit very small unphysical peaks in the range *r* < 1 Å. Moreover, they are in good agreement with the data obtained on BL04B2 using point detectors, for which a 180 times longer exposure time is required to obtain sufficient statistics. A fit to the first peak in the amorphous silica (Mozzi & Warren, 1969 ▸) is shown by the dashed line in Fig. 2 ▸(*b*). The fit parameter is an Si—O distance of 1.62 ± 0.01 Å with a coordination number of 3.89 ± 0.02, which is consistent with the earlier result (Wright, 1990 ▸). Thus, we have confirmed that an FPD with a short exposure time of 1 min provides sufficient data for PDF analysis. Furthermore, an exposure time of 10 s still provides sufficient data for the PDF analysis of amorphous bulk silica, as shown in Fig. 3 ▸. Thus, *in situ* PDF analysis within 10 s is expected to extend the study of structural changes in disordered materials at SPring-8 by reducing the measurement time from hours to seconds.

## 3. Processing of two-dimensional diffraction data (*PIXIA*)   

Because time-resolved PDF measurements provide many two-dimensional diffraction images, rapid and accurate processing of this huge quantity of information is necessary to extract information on structural changes. Thus, we use the *PIXIA* program for data conversion from two-dimensional images into one-dimensional total scattering patterns. *PIXIA* stands for pixel-based image analyser, and it is dedicated to such data conversion for total scattering data. Similar to other programs such as *Fit2D* (Hammersley, 1997 ▸) used for diffractometry, *PIXIA* uses equations applicable over a wide *Q* range suitable for PDF studies, and moreover adopts noise filtering based on an image-processing algorithm. Roughly speaking, *PIXIA* deals with all the pixels of a two-dimensional detector as an array of point detectors to determine a total scattering pattern that corresponds to millions of point detectors (*cf.* 2000 × 2000 pixels). In contrast with conventional programs that perform simple integrations to increase the signal-to-noise ratio, *PIXIA* adopts a mathematical noise-filtration algorithm in different data dimensions.

The *PIXIA* program was written in Python and uses the *SciPy* and *NumPy* packages, in which all the mathematical treatments are dealt with in matrix calculations. This program is a part of the *Orochi* program suite, which is an ensemble of Python-based programs for X-ray diffraction studies including image processing, PDF conversion and data fitting. In this study, we used *PIXIA* (β version 2.x), but the latest version, β version 3.x, can work with the *MaterialsPDF* PDF conversion program in the *Orochi* suite, and can monitor PDFs in real time during experiments. Such a demonstration will be reported in the future. The program will be available free of charge and is currently under testing as a β version available from the website of the National Institute for Materials Science (Tominaka, https://samurai.nims.go.jp/profiles/tominaka_satoshi).

**Figure 4:**
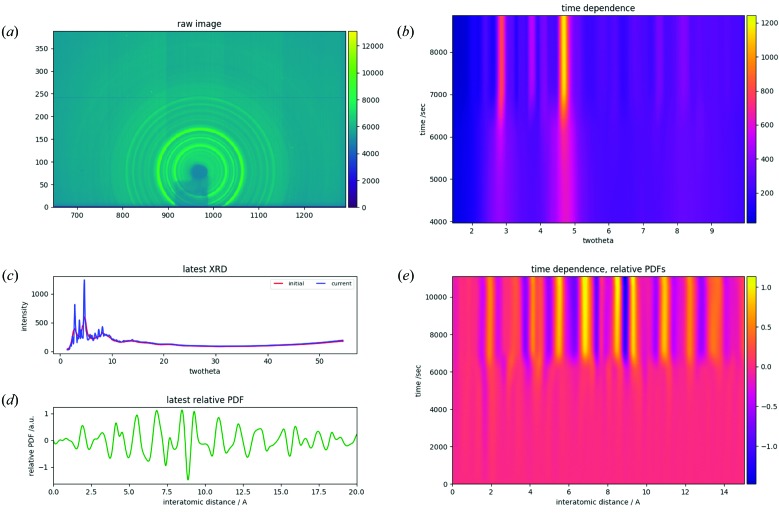
The main graphical user interface of the data-analysis software (*PIXIA*), showing results for the Li_7_P_3_S_11_ sulfide glass. (*a*) The raw two-dimensional image data. (*b*) The time dependence of the total scattering data as an *I*(θ, *t*) plot. (*c*) A comparison of initial and latest total scattering patterns. (*d*) The latest relative PDF calculated based on the initial PDF. (*e*) The time dependence of the relative PDFs.

The *Orochi* program suite has a graphical user interface written in wxPython and data are illustrated using *Matplotlib*. Examples of plots of data treated using *PIXIA* are shown in Fig. 4 ▸: panel (*a*) illustrates the raw two-dimensional image data; panel (*b*) illustrates the time dependence of the total scattering data as an *I*(θ, *t*) plot; panel (*c*) compares initial and latest total scattering patterns; panel (*d*) illustrates the latest relative PDF calculated based on the initial PDF; and panel (*e*) illustrates the time dependence of the relative PDFs. These data were analysed and plotted within a short time simultaneously with the measurements, *i.e.* substantially in real time. This enables us to discuss the data in real time and optimize the measurements taken in a period of beam time.

## 4. Observation of crystallization of sulfide glass during annealing   

The sulfide glass Li_7_P_3_S_11_ is a very important material for all-solid-state Li-ion batteries, in which it is used as a solid electrolyte. Its Li ionic conductivity increases upon thermal crystallization (Mizuno *et al.*, 2005 ▸) and, intriguingly, the conductivity depends on the annealing conditions such as the annealing rate. Thus, we investigated its structural changes upon thermal crystallization at two different annealing rates (2 and 10 K min^−1^). The time-dependent data obtained at the annealing rate of 2 K min^−1^ are shown in Fig. 4 ▸. As can be seen in Fig. 4 ▸(*e*), the relative PDF indicates a clear structural change after 6000 s, which corresponds to a sample temperature of 523 K (Fig. S1 in the supporting information). This is the typical crystallization temperature of the sulfide.

**Figure 5:**
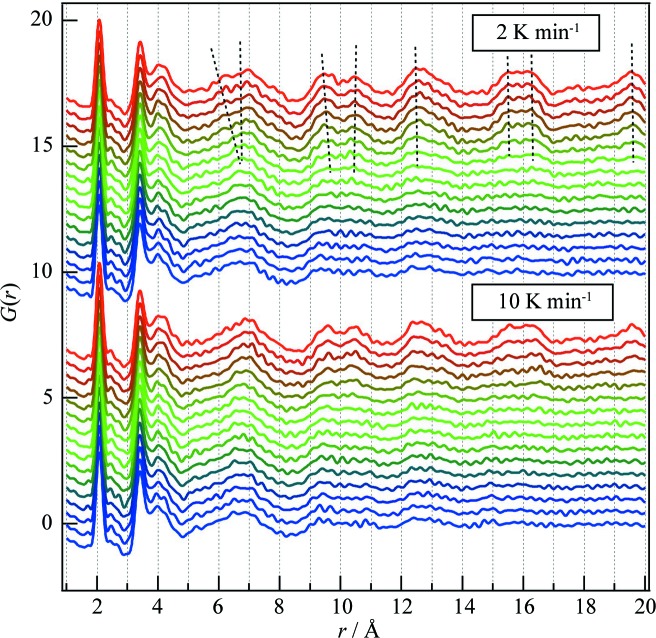
Reduced PDFs *G*(*r*) of Li_7_P_3_S_11_ used to investigate its thermal crystallization. The data were collected in the temperature range from 473 to 573 K at annealing rates of 2 and 10 K min^−1^.

To determine the effect of such annealing on the structure of the sulfide glass at the atomic and molecular levels, time-resolved PDFs, collected in the temperature range from 473 to 573 K at 5 K intervals as shown in Fig. 5 ▸, were analysed. The first peak around 2.0 Å is assigned to the P—S bonds in the PS_4_ tetrahedral anions. This peak does not depend on the annealing treatment, meaning that the PS_4_ tetrahedral anions remain in the structure regardless of the crystallization temperature. On the other hand, the relative intensities of the peaks found at longer *r* (3.4, 4.0, 6.8, 9.6, 10.6, 12.5, 15.5, 16.2 and 19.6 Å) gradually change upon crystallization, as shown by dashed lines in Fig. 5 ▸.

**Figure 6:**
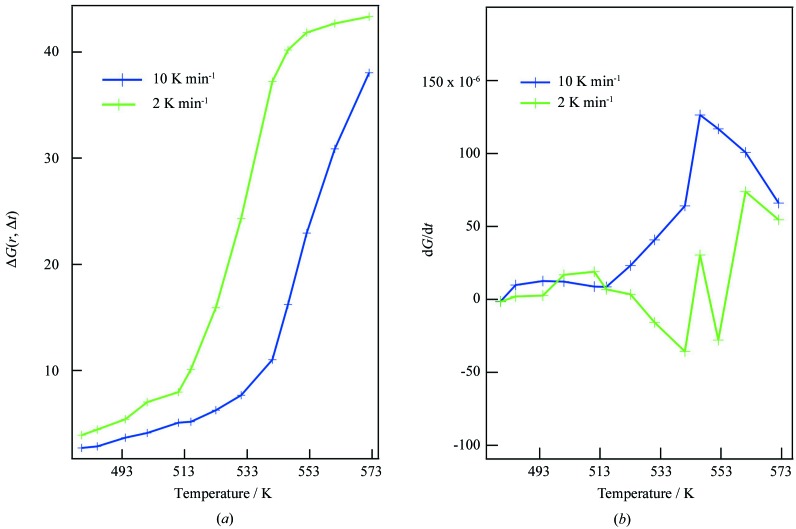
Rate law of structural changes in Li_7_P_3_S_11_ in the temperature range from 473 to 573 K at annealing rates of 2 and 10 K min^−1^. (*a*) Δ*G*(*r*, Δ*t*) was calculated as the difference from *G*(*r*) at 373 K in the *r* range of 15–17 Å. (*b*) d*G*/d*t* was calculated by differentiating Δ*G*(*r*, Δ*t*).

Although different structural changes were expected in the samples annealed at different rates, no clear difference was observed, except for the crystallization temperature: the structural changes commenced at a lower temperature in the sulfide annealed at 2 K min^−1^ than in the sample annealed at 10 K min^−1^. This difference is clear in PDFs obtained at longer *r*, as can be seen from the peaks at *r* = 15.5, 16.2 and 19.6 Å. Moreover, a peak around 6 Å, which is attributed to the formation of Li_4_P_2_S_6_ (Sadowski *et al.*, 2018 ▸), is observed at 546 K only in the case of annealing at 2 K min^−1^. Such decomposition of Li_7_P_3_S_11_ into Li_4_P_2_S_6_ was also reported by Busche *et al.* (2016 ▸) and Minami *et al.* (2010 ▸). Δ*G*(*r*, Δ*t*) was calculated as the difference from *G*(*r*) at 373 K in the *r* range of 15–17 Å (Fig. 6 ▸ *a*). To discuss the kinetics, we plot the relationship between d*G*/d*t* and temperature in Fig. 6 ▸(*b*). d*G*/d*t* was calculated by differentiating Δ*G*(*r*, Δ*t*). As can be seen in this figure, the crystallization of Li_7_P_3_S_11_ was facilitated at around 546 K in the 10 K min^−1^ case. This suggests that crystallization proceeds through the formation of sulfur-deficient fragments of P_2_S_6_ anions as an intermediate between the amorphous phase and the crystalline phase, both of which consist of PS_4_ tetrahedral anions. Further detailed investigations will be reported in the future.

These results demonstrate that the new apparatus for time-resolved PDF analysis on BL08W and BL04B2 can collect PDF data for amorphous and disordered crystalline materials in real time. The acquisition times of 10–60 s per frame enable the investigation of structural changes in disordered materials in real time with high accuracy.

## 5. Conclusions   

The dedicated setups on beamlines BL04B2 and BL08W, in which a large two-dimensional flat-panel detector is used for time-resolved PDF analysis, have been described in this paper. The data for amorphous silica collected on the two-dimensional detector in a few seconds were consistent with the data collected on a point detector scanned for 2–3 h in the existing PDF apparatus on BL04B2. In addition, the apparatus used for time-resolved PDF analysis is compatible with existing instruments for different sample environments, such as an aerodynamic levitation furnace, an acoustic levitation furnace and a diamond anvil cell. To obtain a better understanding of the annealing behaviour of Li_7_P_3_S_11_ sulfide glass, time-resolved PDFs were observed at intervals of 5 K. The sulfide glass annealed at 10 K min^−1^ crystallized faster than that annealed at 2 K min^−1^. This might be associated with the formation of sulfur defects, which were observed only in the crystallization at 2 K min^−1^. Further detailed investigations will be reported in the future.

It has been demonstrated that the new apparatus for time-resolved PDF analysis on BL08W and BL04B2 can collect PDF data for amorphous and disordered crystalline materials in real time. We believe that many structural studies on disordered materials by time-resolved PDF analysis will be performed at SPring-8 using the new apparatus.

## Supplementary Material

Fig. S1: Variation of temperature with time at different annealing rates. DOI: 10.1107/S1600577518011232/il5011sup1.pdf

## Figures and Tables

**Table 1:** Comparison of two beamlines for high-energy X-ray diffraction at SPring-8

Beamline	BL04B2	BL08W
Energy (keV)	61.4	115.6
Energy resolution	5 × 10^−3^	1.5 × 10^−3^
Photon flux (photons s^−1^ mm^−2^)	9.1 × 10^10^	1.0 × 10^13^
*Q*_max_, d*Q* (Å^−1^) (camera length 300 mm)	25, 0.02	50, 0.04
*Q*_max_, d*Q* (Å^−1^) (camera length 800 mm)	12, 0.01	25, 0.02

## References

[bb1] Abdala, P. M., Mauroy, H. & van Beek, W. (2014). *J. Appl. Cryst.* **47**, 449–457.

[bb2] Akola, J., Kohara, S., Ohara, K., Fujiwara, A., Watanabe, Y., Masuno, A., Usuki, T., Kubo, T., Nakahira, A., Nitta, K., Uruga, T., Weber, J. K. R. & Benmore, C. J. (2013). *Proc. Natl Acad. Sci. USA*, **110**, 10129–10134. 10.1073/pnas.1300908110 PMC3690860 23723350

[bb3] Benmore, C. J. (2012). *ISRN Mater. Sci.* **2012**, 852905.

[bb4] Billinge, S. J. L. & Levin, I. (2007). *Science*, **316**, 561–565. 10.1126/science.1135080 17463280

[bb5] Busche, M. R., Weber, D. A., Schneider, Y., Dietrich, C., Wenzel, S., Leichtweiss, T., Schröder, D., Zhang, W., Weigand, H., Walter, D., Sedlmaier, S. J., Houtarde, D., Nazar, L. F. & Janek, J. (2016). *Chem. Mater.* **28**, 6152–6165.

[bb6] Chupas, P. J., Chapman, K. W. & Lee, P. L. (2007). *J. Appl. Cryst.* **40**, 463–470. 10.1107/S0021889806047923 PMC2483494 19461850

[bb7] Chupas, P. J., Chaudhuri, S., Hanson, J. C., Qiu, X., Lee, P. L., Shastri, S. D., Billinge, S. J. L. & Grey, C. P. (2004). *J. Am. Chem. Soc.* **126**, 4756–4757. 10.1021/ja031553n 15080661

[bb8] Chupas, P. J., Qiu, X., Hanson, J. C., Lee, P. L., Grey, C. P. & Billinge, S. J. L. (2003). *J. Appl. Cryst.* **36**, 1342–1347.

[bb9] Hammersley, A. P. (1997). ESRF Internal Report ESRF97HA02T. ESRF, Grenoble, France.

[bb10] Inui, M., Matsuda, K., Ishikawa, D., Tamura, K. & Ohishi, Y. (2007). *Phys. Rev. Lett.* **98**, 185504. 10.1103/PhysRevLett.98.185504 17501585

[bb11] Isshiki, M., Ohishi, Y., Goto, S., Takeshita, K. & Ishikawa, T. (2001). *Nucl. Instrum. Methods Phys. Res. A*, **467–468**, 663–666.

[bb12] Jensen, K. M. Ø., Christensen, M., Juhas, P., Tyrsted, C., Bøjesen, E. D., Lock, N., Billinge, S. J. L. & Iversen, B. B. (2012). *J. Am. Chem. Soc.* **134**, 6785–6792. 10.1021/ja300978f 22420861

[bb13] Keen, D. (2001). *J. Appl. Cryst.* **34**, 172–177.

[bb14] Keen, D. A. & Goodwin, A. L. (2015). *Nature (London)*, **521**, 303–309. 10.1038/nature14453 25993960

[bb15] Keen, D. A., Keeble, D. S. & Bennett, T. D. (2018). *Phys. Chem. Miner.* **45**, 333–342.

[bb16] Kohara, S., Akola, J., Patrikeev, L., Ropo, M., Ohara, K., Itou, M., Fujiwara, A., Yahiro, J., Okada, J. T., Ishikawa, T., Mizuno, A., Masuno, A., Watanabe, Y. & Usuki, T. (2014). *Nat. Commun.* **5**, 5892. 10.1038/ncomms6892 PMC4284809 25520236

[bb17] Kohara, S., Itou, M., Suzuya, K., Inamura, Y., Sakurai, Y., Ohishi, Y. & Takata, M. (2007). *J. Phys. Condens. Matter*, **19**, 506101.

[bb18] Kohara, S., Suzuya, K., Takeuchi, K., Loong, C. K., Grimsditch, M., Weber, J. K. R., Tangeman, J. A. & Key, T. S. (2004). *Science*, **303**, 1649–1652. 10.1126/science.1095047 15016995

[bb19] Kramer, M. (2007). *J. Appl. Cryst.* **40**, 77–86.

[bb20] Li, B., Wang, H., Kawakita, Y., Zhang, Q., Feygenson, M., Yu, H. L., Wu, D., Ohara, K., Kikuchi, T., Shibata, K., Yamada, T., Ning, X. K., Chen, Y., He, J. Q., Vaknin, D., Wu, R. Q., Nakajima, K. & Kanatzidis, M. G. (2018). *Nat. Mater.* **17**, 226–230. 10.1038/s41563-017-0004-2 29335610

[bb21] Liu, Z., Okabe, K., Anand, C., Yonezawa, Y., Zhu, J., Yamada, H., Endo, A., Yanaba, Y., Yoshikawa, T., Ohara, K., Okubo, T. & Wakihara, T. (2016). *Proc. Natl Acad. Sci. USA*, **113**, 14267–14271. 10.1073/pnas.1615872113 PMC5167192 27911823

[bb22] Lorch, E. (1969). *J. Phys. C Solid State Phys.* **2**, 229–237.

[bb23] Maréchal, X.-M., Hara, T., Tanabe, T., Tanaka, T. & Kitamura, H. (1998). *J. Synchrotron Rad.* **5**, 431–433. 10.1107/S0909049597017081 15263535

[bb24] Matsumoto, T., Abe, T., Furukawa, Y., Masunaga, H., Matsushita, T. & Nakada, K. (2018). *Proceedings of the 16th International Conference on Accelerator and Large Experimental Control Systems (ICALEPCS2017)*, 8–13 October 2017, Barcelona, Spain, pp. 1281–1285. THMPL07.

[bb25] Matsunaga, T., Akola, J., Kohara, S., Honma, T., Kobayashi, K., Ikenaga, E., Jones, R. O., Yamada, N., Takata, M. & Kojima, R. (2011). *Nat. Mater.* **10**, 129–134. 10.1038/nmat2931 21217690

[bb26] Minami, K., Hayashi, A. & Tatsumisago, M. (2010). *J. Ceram. Soc. Jpn*, **118**, 305–308.

[bb27] Mizuno, F., Hayashi, A., Tadanaga, K. & Tatsumisago, M. (2005). *Adv. Mater.* **17**, 918–921.

[bb28] Mozzi, R. L. & Warren, B. E. (1969). *J. Appl. Cryst.* **2**, 164–172.

[bb29] Ohara, K., Temleitner, L., Sugimoto, K., Kohara, S., Matsunaga, T., Pusztai, L., Itou, M., Ohsumi, H., Kojima, R., Yamada, N., Usuki, T., Fujiwara, A. & Takata, M. (2012). *Adv. Funct. Mater.* **22**, 2251–2257.

[bb30] Ohno, H., Kohara, S., Umesaki, N. & Suzuya, K. (2001). *J. Non-Cryst. Solids*, **293–295**, 125–135.

[bb31] Onodera, Y., Kohara, S., Masai, H., Koreeda, A., Okamura, S. & Ohkubo, T. (2017). *Nat. Commun.* **8**, 15449. 10.1038/ncomms15449 PMC5499210 28561027

[bb32] Sadowski, M., Sicolo, S. & Albe, K. (2018). *Solid State Ion.* **319**, 53–60.

[bb33] Terban, M. W., Johnson, M., Di Michiel, M. & Billinge, S. J. L. (2015). *Nanoscale*, **7**, 5480–5487. 10.1039/c4nr06486k 25732228

[bb34] Tominaka, S., Hiroki, Y., Satoshi, H., Saori , I. K. & Koji, O. (2018). *ACS Omega*, **3**, 8874–8881.

[bb35] Tominaka, S., Kawakami, K., Fukushima, M. & Miyazaki, A. (2017). *Mol. Pharm.* **14**, 264–273. 10.1021/acs.molpharmaceut.6b00866 28043129

[bb36] Umeda, T., Yamada, H., Ohara, K., Yoshida, K., Sasaki, Y., Takano, M., Inagaki, S., Kubota, Y., Takewaki, T., Okubo, T. & Wakihara, T. (2017). *J. Phys. Chem. C*, **121**, 24324–24334.

[bb37] Wright, A. C. (1990). *J. Non-Cryst. Solids*, **123**, 129–148.

[bb38] Yoneda, Y., Kohara, S., Ito, M., Abe, H., Takeuchi, M., Uchida, H. & Matsumura, Y. (2013). *Trans. Mater. Res. Soc. Jpn*, **38**, 109–112.

[bb39] Young, C. A. & Goodwin, A. L. (2011). *J. Mater. Chem.* **21**, 6464–6476.

