# Supplementary material for: Time-resolved pair distribution function analysis of disordered materials on beamlines BL04B2 and BL08W at SPring-8
Source: J Synchrotron Radiat. 2018 Sep 26;25(Pt 6):1627–33. doi: 10.1107/S1600577518011232 (PMC6225740; doi:10.1107/S1600577518011232)
Supplement: Supplementary file 1 [file s-25-01627-sup1.pdf]

JOURNAL OF SYNCHROTRON RADIATION

**Volume 25 (2018)**

**Supporting information for article:**

# Time-resolved pair distribution function analysis of disordered materials on beamlines BL04B2 and BL08W at SPring-8

**Koji Ohara, Satoshi Tominaka, Hiroki Yamada, Masakuni Takahashi, Hiroshi Yamaguchi, Futoshi Utsuno, Takashi Umeki, Atsushi Yao, Kengo Nakada, Michitaka Takemoto, Satoshi Hiroi, Naruki Tsuji and Toru Wakihara**

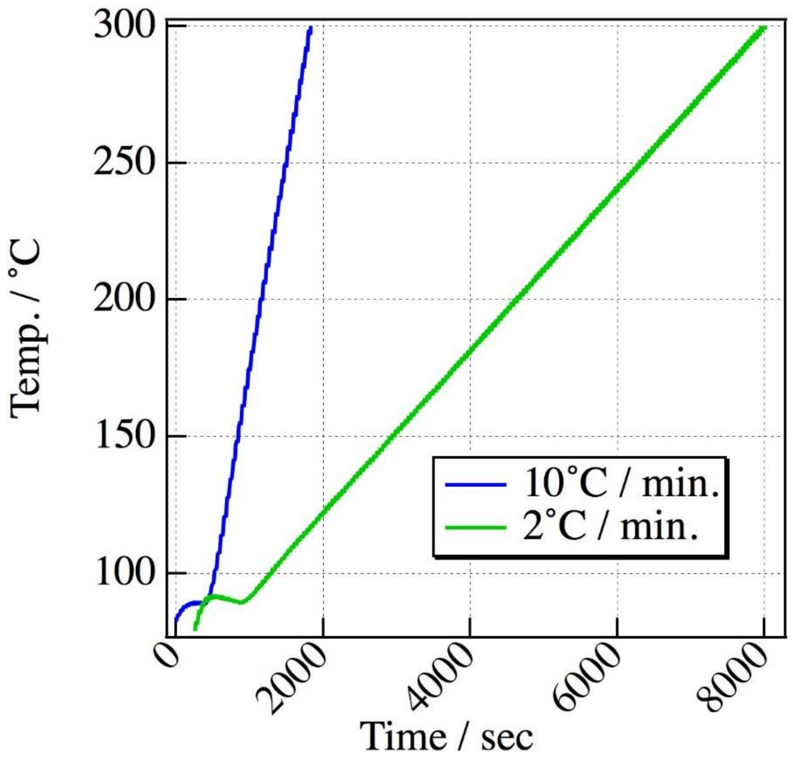


**Figure S1** Variations of temperature with time for annealing rates of 2 and 10 °C/min.
